# Filaments of light dynamically isolate a single attosecond pulse

**DOI:** 10.1038/s41467-026-71478-w

**Published:** 2026-04-16

**Authors:** Omair Ghafur

**Affiliations:** Department of Physics, The Ohio State University, Columbus, OH USA

**Keywords:** Optical techniques, Ultrafast lasers, High-harmonic generation, High-harmonic generation, Nonlinear optics

## Abstract

By matching a driving laser’s peak power to the self-focusing threshold in an extended gas cell, a nonlinear light filament is formed that naturally isolates attosecond pulses. As it propagates, the filament autonomously shortens and spatially cleans the driving beam, while simultaneously altering the local medium properties to synchronize the light fields. This dynamic effect restricts the coherent buildup of emitted radiation to a single half-cycle, eliminating the need for complex external optical gating.

Observing fundamental processes in real-time, from breaking chemical bonds^1^ to electron dynamics^2–4^, requires extraordinarily short pulses of light—as brief as tens of attoseconds (1 as = 10^−18^ s). Such attosecond pulses are typically produced via high-harmonic generation (HHG), a process in which an intense laser field ionizes atoms in a gas target and drives recollision of the freed electrons to produce bursts of extreme-ultraviolet (XUV) light on each half-cycle of the driving field. The resulting train of attosecond pulses enables new precision measurements, such as the time delay of photoionization^2^.

However, both the need to track non-repeating dynamics and the broad bandwidth of isolated pulses make their generation a central challenge. This is conventionally tackled with elaborate optical gating schemes such as polarization gating^5^, double optical gating^6^ or ionization gating^7^. These schemes demand precisely aligned waveplates, interferometers, and few-cycle driver pulses whose carrier-envelope phase must be stabilized—all to confine HHG emission to a single half-cycle^8^. This complexity has restricted isolated attosecond pulses to a handful of specialized laboratories.

Now writing in *Nature Communications*, Chien et al.^9^ bypass the complexity of traditional optical gating by demonstrating the generation of isolated attosecond pulses through laser filamentation—a high-intensity self-guided channel of light unique to ultrashort pulses. The method is deeply reminiscent of the serendipitous discovery of Kerr-lens mode-locking in the 1990s^10,11^, where the Kerr effect acted as a spatial filter that sustained ultrashort pulses in laser oscillators. This self-organizing nonlinearity once unlocked the femtosecond domain. Now, it opens a path to the attosecond frontier.

In a conventional cell, a focused pulse hits a wall of gas, and the resulting plasma distorts the beam almost instantly. In a semi-infinite gas cell, a density gradient lets nonlinearities develop gradually. Over this drawn-out path, the Kerr effect induces spatial self-focusing (see Fig. 1). Left unchecked, this would cause catastrophic beam collapse. However, the rising intensity ionizes the gas, creating a free-electron plasma that acts as a diverging lens to counter the Kerr focusing. This tug-of-war forms an extended filament of light, clamping the peak intensity near the ionization threshold^12^.

**Fig. 1:**
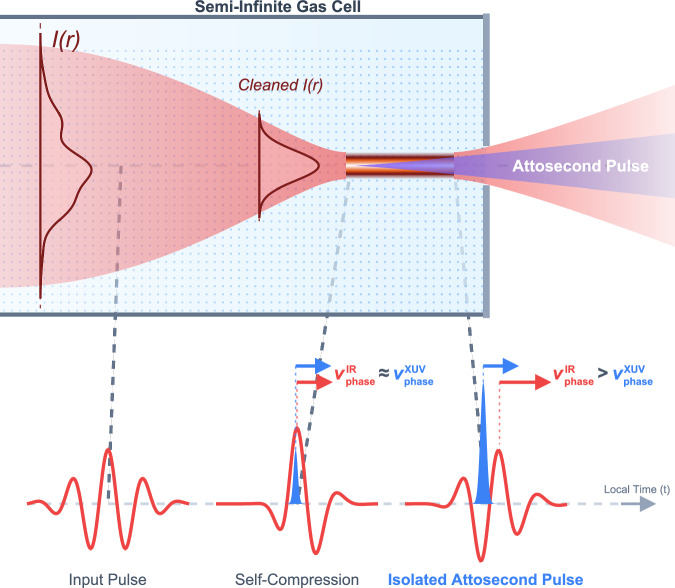
Spatiotemporal self-gating in a semi-infinite gas cell. An infrared driving pulse (red, Input Pulse) with an irregular intensity profile, *I*(*r*), enters an extended gas medium. The dynamic balance between Kerr self-focusing and plasma defocusing induces filamentation, which acts simultaneously as a spatial filter and as a temporal compressor (Self-Compression). At the pulse peak, the Kerr and plasma contributions to the refractive index cancel, synchronizing the phase velocities of the driving infrared field and the emitted radiation (${v}_{{{\rm{phase}}}}^{\,{{\rm{IR}}}}\approx {v}_{{{\rm{phase}}}}^{\,{{\rm{XUV}}}}$). This opens a transient phase-matching window for coherent XUV buildup. Immediately following the peak, the increase in free-electron plasma density accelerates the infrared phase velocity beyond the speed of light in vacuum (${v}_{{{\rm{phase}}}}^{\,{{\rm{IR}}}} > {v}_{{{\rm{phase}}}}^{\,{{\rm{XUV}}}}$). This rapid phase advance dephases all trailing emission, restricting XUV generation to a single half-cycle—producing an Isolated Attosecond Pulse.

More importantly, this mechanism creates a transient phase-matching window for HHG. Coherent build-up of XUV light requires that the generated radiation and the driving infrared field share the same phase velocity. For a pulse undergoing filamentation, neutral gas dispersion and the Kerr effect slow the infrared wave on the leading edge, while rapid plasma generation accelerates its phase velocity beyond the speed of light on the trailing edge. Only at the exact peak of the pulse do these effects perfectly cancel. This fleeting synchronization acts as an ultrafast shutter, spontaneously restricting efficient generation to a single half-cycle of the driver.

This self-organizing gate provides a higher temporal contrast than amplitude gating alone, yielding near-transform-limited isolated attosecond pulses of 65 attoseconds duration at a photon energy of ≈135 eV in helium^9^. Compared to the group’s previous work with 3.1 fs pulses, where satellite pulses were produced at 25% relative intensity^13^, the filamentation-assisted approach suppresses them almost entirely.

However, the very self-organizing character that simplifies the apparatus also limits when it can be used. Filamentation requires the input peak power to remain near the critical power for self-focusing. Attempting to scale HHG yield to driving pulses with higher energy breaks the beam into multiple filaments, destroying the conditions for clean HHG^9,12,14^. This constrains the technique’s applicability to laser systems where the average power scales with the repetition rate rather than pulse energy. An extension to longer wavelength driving pulses—needed to access the water-window regime^15^—is equally challenging, as the critical power is proportional to the square of the wavelength, demanding driving powers that are proportionally higher. Longer wavelengths may also shift the Kerr-plasma balance in ways that could suppress spatial cleaning and self-compression.

Fundamentally, a limitation of the self-organizing gate is the loss of deterministic control. Spatial filtering, pulse compression, and phase matching are determined by the nonlinear dynamics in the gas, and they cannot be tuned independently. Thus, to adjust the photon energy, Chien et al.^9^ must change to a different gas medium. Simplicity has been bought. Control was the price.

However, this surrender of control does have some practical appeal. With this work, the complexity of more conventional optical gating setups has been transferred to a set of continuously adjustable parameters, including gas pressure, focal position, iris aperture, and glass wedge insertion—variables that are all easily automated and targeted by machine-learning optimization^16^.

Three decades after the Kerr effect broke the femtosecond barrier, it has now, balanced against a plasma, organized itself into an attosecond gate. Whether this technique will become as ubiquitous as the Kerr-lens mode-locked oscillator remains to be seen, but it serves as a reminder that in nonlinear optics, the medium sometimes knows best.

## References

[1] Zewail, A. H. Nobel Lecture: Femtochemistry: Atomic-scale dynamics of the chemical bond using ultrafast lasers. *Rev. Mod. Phys.* **72**, 1043–1065 (2000). 10.1002/1521-3773(20000804)39:15<2586::aid-anie2586>3.0.co;2-o 10934390

[2] L’Huillier, A. Nobel Lecture: The route to attosecond pulses. *Rev. Mod. Phys.* **96**, 030503 (2024).

[3] Krausz, F. Nobel Lecture: Sub-atomic motions. *Rev. Mod. Phys.* **96**, 030502 (2024).

[4] Agostini, P. Nobel Lecture: Genesis and applications of attosecond pulse trains. *Rev. Mod. Phys.* **96**, 030501 (2024).

[5] Sansone, G. et al. Isolated single-cycle attosecond pulses. *Science* **314**, 443–446 (2006). 17053142 10.1126/science.1132838

[6] Mashiko, H. et al. Double optical gating of high-order harmonic generation with carrier-envelope phase stabilized lasers. *Phys. Rev. Lett.* **100**, 103906 (2008). 18352191 10.1103/PhysRevLett.100.103906

[7] Ferrari, F. et al. High-energy isolated attosecond pulses generated by above-saturation few-cycle fields. *Nat. Photonics* **4**, 875–879 (2010).

[8] Midorikawa, K. Progress on table-top isolated attosecond light sources. *Nat. Photonics* **16**, 267–278 (2022).

[9] Chien, Y.-E. et al. Filamentation-assisted isolated attosecond pulse generation. *Nat. Commun.* ArXiv:2412.06339 [physics.optics]. (2024). 10.1038/s41467-026-70903-4 PMC13087264 41991917

[10] Spence, D. E., Kean, P. N. & Sibbett, W. 60-fsec pulse generation from a self-mode-locked ti:sapphire laser. *Opt. Lett.* **16**, 42–44 (1991). 19773831 10.1364/ol.16.000042

[11] Brabec, T., Spielmann, C., Curley, P. F. & Krausz, F. Kerr lens mode locking. *Opt. Lett.* **17**, 1292–1294 (1992). 19798161 10.1364/ol.17.001292

[12] Couairon, A. & Mysyrowicz, A. Femtosecond filamentation in transparent media. *Phys. Rep.* **441**, 47–189 (2007).

[13] Tsai, M.-S. et al. Nonlinear compression toward high-energy single-cycle pulses by cascaded focus and compression. *Sci. Adv.* **8**, eabo1945 (2022). 35921417 10.1126/sciadv.abo1945 PMC9348793

[14] Heyl, C. M., Arnold, C. L., Couairon, A. & L’Huillier, A. Introduction to macroscopic power scaling principles for high-order harmonic generation. *J. Phys. B: At., Mol. Opt. Phys.* **50**, 013001 (2017).

[15] Corkum, P. B. Plasma perspective on strong field multiphoton ionization. *Phys. Rev. Lett.* **71**, 1994–1997 (1993). 10054556 10.1103/PhysRevLett.71.1994

[16] Genty, G. et al. Machine learning and applications in ultrafast photonics. *Nat. Photonics* **15**, 91–101 (2021).

